# Strategies for combating antibiotic resistance in bacterial biofilms

**DOI:** 10.3389/fcimb.2024.1352273

**Published:** 2024-01-19

**Authors:** Kayla E. Grooters, Jennifer C. Ku, David M. Richter, Matthew J. Krinock, Ashley Minor, Patrick Li, Audrey Kim, Robert Sawyer, Yong Li

**Affiliations:** ^1^ Department of Medicine, Western Michigan University Homer Stryker M.D. School of Medicine, Kalamazoo, MI, United States; ^2^ University of Michigan, Ann Arbor, MI, United States; ^3^ Division of Biomedical Engineering, Department of Orthopedic Surgery, Western Michigan University Homer Stryker M.D. School of Medicine, Kalamazoo, MI, United States; ^4^ Department of Surgery, Western Michigan University Homer Stryker M.D. School of Medicine, Kalamazoo, MI, United States

**Keywords:** biofilm, infection, bacteriophage, antibiotic resistance, treatment

## Abstract

Biofilms, which are complexes of microorganisms that adhere to surfaces and secrete protective extracellular matrices, wield substantial influence across diverse domains such as medicine, industry, and environmental science. Despite ongoing challenges posed by biofilms in clinical medicine, research in this field remains dynamic and indeterminate. This article provides a contemporary assessment of biofilms and their treatment, with a focus on recent advances, to chronicle the evolving landscape of biofilm research.

## 1 Introduction

A biofilm is an immobile, three-dimensional matrix of microscopic organisms that have aggregated onto a surface to form a colony (Sharma et al., 2019). The organisms secrete adhesive proteins and extracellular matrix which help cement the cells to a surface and protect the colony from decussation, environmental hazards, host defenses, and antimicrobial compounds (Jacqueline and Caillon, 2014). One of the key issues with using antibiotics to treat biofilms is achieving the required minimum inhibitory concentration (MIC) of drug at the infection site. The MIC for a biofilm can be between 100-800x greater than the MIC for planktonic cells (Jacqueline and Caillon, 2014). In addition, singular bacteria within biofilms that have been exposed to high concentrations of antibiotics can persist and reestablish a more resistant biofilm, a phenomenon known as recalcitrance (Ciofu et al., 2022). Consequentially, biofilms are frequently refractory to antibiotic treatment and, thus, may require surgical intervention. However, surgery may still prove ineffective, resulting in significant morbidity and mortality, with biofilms implicated in over 500,000 deaths per year in the United States alone (Charani and Holmes, 2019).

Biofilms are known to occur in every human organ system, ranging from the respiratory and digestive tracts to the heart, eyes, and ears (Perry and Tan, 2023). Indeed, biofilms have been implicated in 65% of all bacterial infections (Jamal et al., 2018) and nearly 80% of chronic wounds (Malone et al., 2017). Interestingly, the incidence of biofilm-associated infections is on the rise (Cámara et al., 2022). Many such biofilms exhibit resistance to typical antibiotics and, thus, delay healing time and may require invasive interventions to resolve infection (Metcalf and Bowler, 2013). Furthermore, biofilms present important challenges for the design and use of invasive medical products and prosthetics. For example, biofilms are frequently implicated in catheter-associated infections (Gominet et al., 2017), where they complicate decontamination and treatment of the infection (Ielapi et al., 2020). Biofilms present similar complications in other life-saving interventions, such as endotracheal intubation (Diaconu et al., 2018). Importantly, biofilms commonly affect implanted devices—such as prosthetic joints and pacemakers—and are frequently refractory to pharmacological treatment, ultimately requiring removal of the device (Santos et al., 2011; Tande and Patel, 2014). As a result, recent analyses have estimated the global impact of biofilms to be upwards of $280 billion (Cámara et al., 2022).

Given such significant human and financial costs, there is an increasingly urgent need to develop novel strategies for the clinical management of biofilms. In this review, we focus on the formation and structure of biofilms, the mechanisms of antibiotic resistance within these systems, and highlight emerging non-antibiotic mechanisms of biofilm control.

## 2 Formation of bacterial biofilms

**Figure 1:**
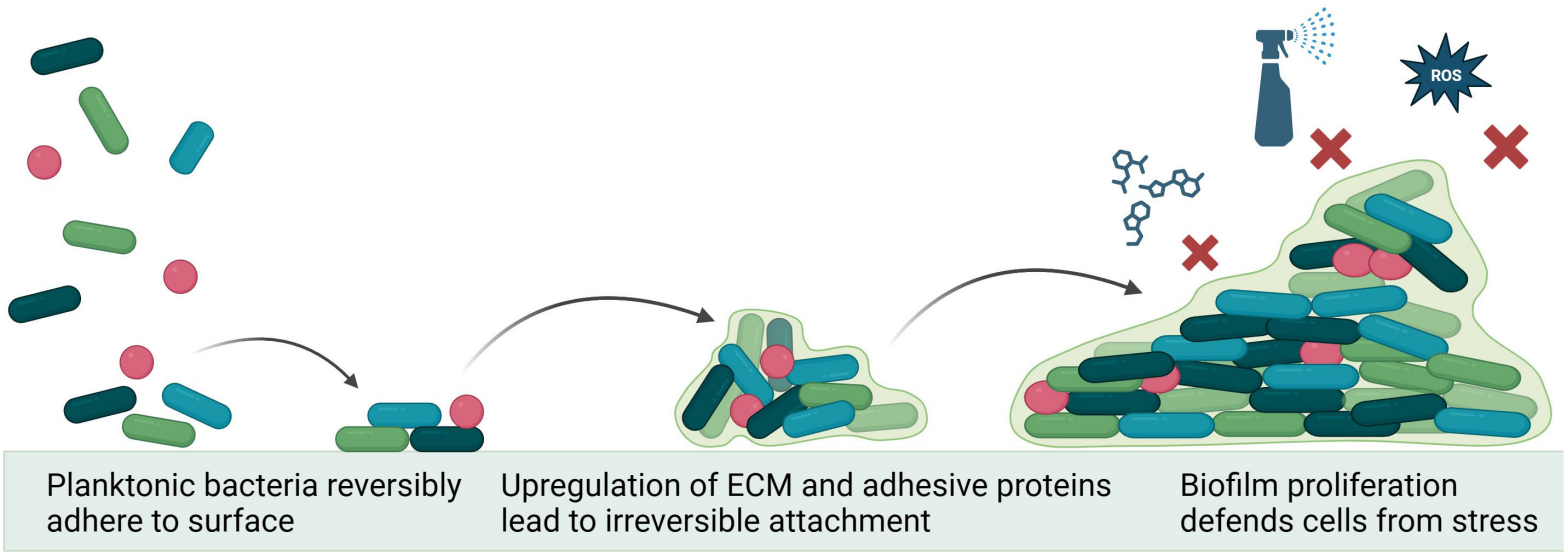
Mechanism of biofilm formation. Environmental conditions lead planktonic bacteria to utilize adhesion machinery to attach to a surface. Quorum sensing between colony members drives upregulation of extracellular matrix formation and changes in metabolic function, irreversibly cementing biofilm to surface and protecting the colony from environmental hazards (e.g., antiseptics, reactive oxygen species (ROS) and antibiotics. Figure made using Biorender.com.

Biofilm formation is initiated by a complex series of environmental and genetic triggers, primarily involved in stress responses. External factors such as pH, temperature, nutrient availability, and environmental hazards all play a role in causing a planktonic microorganism to shift into an adherent state (Rather et al., 2021). The first step of biofilm formation is reversible adherence, where microorganisms use attachment devices, such as flagella, pili, and fimbriae, to glue themselves to an available substrate. During this stage, the microorganisms are free to abandon their attachment site and return to planktonic life or commit to irreversible attachment (Toyofuku et al., 2016). During irreversible attachment, the microorganisms upregulate various adhesion molecules and glycoproteins. From here, cells undergo division and microcolony formation. Bacteria in the colony communicate through quorum sensing, a process dependent on the synthesis, detection, and regulation of autoinducing molecules (**Figure 1**). This communication directs the rate of cell division and production of extracellular polymeric substance (Asma et al., 2022)—which accounts for over 90% of the dry mass of mature biofilms (Toyofuku et al., 2016).

### 2.1 Environmental control

Specifically, a biofilm’s extracellular polymeric substance (EPS) matrix, which is composed of proteins, polysaccharides, extracellular DNA, and lipids, allows it to withstand challenges like fluid shear and mechanical pressure. While increased EPS production can only be speculated for environmental challenges like fluid shear, it was found that in staphylococcal biofilms, increased mechanical pressure stimulated the EPS of the biofilm to produce more polysaccharides (Hou et al., 2018). Further, hypoxic conditions may foster formation of bacterial biofilms—particularly for those involving *Staphylococcus aureus*. In the case of *S. aureus* CIP 53.154, hypoxia results in a 21-fold increase in biofilm production, associated with concomitant downregulation of *lexA*—a stress-response-related gene—indicating the hypoxic conditions were favorable for growth (Lamret et al., 2021). Similarly, Aristotelous (2022) found biofilms were unable to thrive in well-oxygenated environments, likely due to enhanced phagocytosis by neutrophils; however, under hypoxic conditions, biofilm-secreted virulence factors decreased the effectiveness of neutrophil phagocytosis and promoted bacterial persistence (Aristotelous, 2022). These studies illustrate how harsh environments—e.g., those with fluid shear, mechanical pressure, and hypoxia—are quite habitable environments for many forms of biofilms.

## 3 Current management and treatment of biofilm infections

The cohesion of microorganisms leading to biofilm formation autonomously generates an extracellular matrix, establishing environments that promote bacterial tolerance and resistance to antibiotics through diverse mechanisms contingent upon factors, such as biofilm composition and prevailing growth conditions. Although many studies have studied drug penetration through the biofilm barrier, the underlying mechanisms remain inconclusive. Thus, understanding the mechanisms underlying biofilms’ contribution to antibiotic tolerance and resistance is crucial for devising innovative strategies to combat these infections.

### 3.1 Structure: density and penetration

The efficacy of biofilm treatment is linked to the ability of the antimicrobial agent to penetrate the heterogenous biofilm structure. It has been shown that the capacity of the drug to penetrate the biofilm is highly dependent upon biofilm structure, bacteria genus and strain, and selected antibiotic (Singh et al., 2016). Extracellular DNA, a constituent of the structural framework of the biofilm, has been demonstrated to induce antibiotic resistance (Panlilio and Rice, 2021). Furthermore, the resistance of biofilms to antibiotics is significantly influenced by the bacterial exopolysaccharide (EPS) matrix, a key component in biofilm formation and maintenance (Liu et al., 2017). The production of EPS serves as an adaptive mechanism, with bacteria synthesizing them under stressful conditions, including exposure to antibiotics (Vazquez-Rodriguez et al., 2018). The reduced penetration through the EPS matrix constitutes a mechanism through which biofilms resist antibiotics (Yasir et al., 2018). Factors affecting antibiotic penetration include increased biofilm thickness, drug diffusion efficacy, and the concentration and duration of the administered antibiotic (Hall and Mah, 2017). Additionally, the slow or incomplete diffusion of antibiotics can trap them within the biofilm, resulting in their inactivation by extracellular matrix enzymes (Pinto et al., 2020).

### 3.2 The metabolic environment within biofilms

The heterogeneity bacterial population observed in biofilms gives rise to metabolically distinct microcolonies. Various mechanisms have been postulated to explain the observed heterogeneity in biofilms. According to the zone model, each bacterium responds to its microenvironment, leading to diverse physiological states within the same biofilm (Kirketerp-Møller et al., 2020). Differences in physiological activity have been shown to be due to differences in pH, hydrogen peroxide, and noncellular materials (Jang et al., 2016; Wu et al., 2018; Ghosh et al., 2019). It has been shown that the deepest layers of the biofilm are exposed to more nutrient-depleted conditions when compared to upper layers of the biofilm due to diffusion barrier and consumption of nutrients carried out by cells in the periphery of the biofilm (Liu et al., 2015). These nutrient-deficient zones have also been identified as a primary source of resistance in bacteria (Liu et al., 2022). Furthermore, nutrient-deficient zones promote the emergence of persister cells, dormant cells that exhibit slow growth and resistance to antibiotics (Olsen, 2015; Miyaue et al., 2018). Therefore, the existence of diverse zones results in a myriad of genotypes and phenotypes coexisting within a local environment. This phenomenon accounts for the emergence of unique metabolic pathways that contribute to drug resistance.

### 3.3 Efflux pumps

Efflux pumps have conventionally been associated with multidrug resistance due to their capability to extrude diverse antibiotics from bacteria (Nishino et al., 2021). Moreover, these pumps are known to play a crucial role in biofilm formation—particularly in the context of biofilm-associated drug assistance. The physiological heterogeneity within biofilms explains the observed patterns of efflux pump gene expression. For instance, Babin et al. noted the upregulation of specific antibiotic resistance pumps in the upper region of biofilms, while downregulation or no change was observed in the middle of the biofilm (Babin et al., 2017). In the case of *Pseudomonas aeruginosa*, it has been demonstrated that hypoxia enhances antibiotic resistance by altering the composition of multidrug efflux pumps (Schaible et al., 2012). Furthermore, efflux pump inhibitors have been shown to block the antibiotic tolerance of biofilms and completely abolish biofilm formation (Kvist et al., 2008; Zimmermann et al., 2019).

### 3.4 Quorum sensing

Biofilm formation is partly regulated by quorum sensing (QS), a mechanism through which bacteria employ signaling molecules to enhance communication and survival (Preda and Săndulescu, 2019). QS has been demonstrated to directly impact the regulation of biofilm resistance to antibiotics; specifically, QS regulates expression of various efflux pumps, subsequently influencing the QS system itself (Wang et al., 2019). Further, QS plays a critical role in formation of both gram-positive and -negative biofilms, albeit through slightly different mechanisms. While gram-negative bacteria employ acyl-homoserine lactones within their QS system, gram-positive bacteria employ larger oligopeptides. Both molecules, however, contribute to biofilm formation, thereby hindering antibiotic penetration (An et al., 2019; Gimza et al., 2019).

## 4 Clinical management of biofilms

The mechanical barrier assembled by biofilms shield constituent microorganisms from antimicrobial agents, thereby presenting significant issues for the clinical management of biofilms. Presently, biofilm management relies on antimicrobial agents and surgical debridement; however, inconsistent treatment outcomes persist. Thus, research in this field is essential to advance the strategies for eradicating biofilms.

### 4.1 Antibacterial therapies

The heterogeneity of biofilm formation presents a significant challenge in biofilm management. While cells within biofilms exhibit a much higher minimum inhibitory concentration of antibiotics, topical administration allows for delivery of elevated antibiotic concentrations to target biolfilms (Overhage et al., 2008; Yang et al., 2017). Antimicrobial agents have shown high efficacy against biofilm-associated bacteria. However, due to antibiotic resistance, combination therapy emerged as a therapeutic strategy for treating biofilm infections. Combining antibiotics with other agents, such as N-acetylcysteine and recombinant deoxyribonuclease I, has been shown to significantly reduce biofilms (Belfield et al., 2017). Furthermore, certain agents, including catechin, protocatechuic, and vanillic acids, exhibit synergistic effects when combined with antibiotics, inhibiting bacterial adhesion and, thus, biofilm formation (Bernal-Mercado et al., 2020). However, the eradication of biofilms using traditional antibiotic therapy remains challenging, as the large doses required to reach a concentration sufficient to eliminate biofilms frequently cause detrimental side effects to the patient (Ciofu et al., 2017).

### 4.2 Surgical debridement

The current best treatment to eradicate biofilms involves surgical debridement (Rodríguez-Merchán et al., 2021). This type of debridement uses sharp instruments to remove non-viable and possibly viable tissue surrounding a wound and requires properly trained medical providers and pain control options (Tran et al., 2023). Surgical debridement allows the wound to be more receptive to antibiotic therapies which increases the likelihood of eradicating the biofilm from the wound (Ousey and Ovens, 2023). While this form of debridement is the standard care for many open wound infections, it is unlikely that complete removal of the biofilm will occur, and new strategies including using surgical debridement with meshed skin graft simultaneously may have better outcomes related to healing and infection rates (Namgoong et al., 2020).

### 4.3 Alternative treatments

Due to the challenges seen with treatments with antibiotics, both as standalone and in combination, research has explored alternative approaches for biofilm eradication. More recently, quaternary ammonium compounds have exhibited high potency and a broad spectrum of activity for biofilm elimination; however, certain analogs have raised concerns regarding toxicity (Saverina et al., 2023). Elevated concentrations of antimicrobial lipids have also been shown potential in eradicating biofilms. In a related study, lipid-coated hybrid nanoparticles were utilized to enhance biofilm penetration for antibiotic treatment (Lee et al., 2022). Additionally, secondary metabolites, such as phenazines and quinolines, have demonstrated complete eradication of certain biofilms with the added benefits of low toxicity; however, it is worth noting that these metabolites have been found to trigger formation of biofilm, dependent on species and strain (Huigens, 2018). For antibiotic resistant biofilms that are challenging to treat, anticancer drugs, such as mitomycin C and cisplatin, have been successfully used as therapies, though clinical toxicity remains a concern (Wakharde et al., 2018). A deeper understanding of these alternative treatments holds potential to pave way for the development of new antibiotics and agents for effective biofilm treatment.

## 5 Novel strategies for eradication

Given the challenges biofilms pose to conventional treatment strategies, there is increasing interest in exploring novel therapeutic therapies. Such strategies aim to exploit various aspects of biofilm—such as the extracellular matrix—without relying on the metabolism of the cells themselves. These techniques are being investigated both for the prevention of biofilm formation on biotic and abiotic surfaces, as well as for the treatment of active infections.

### 5.1 Light-based strategies

The use of Ultraviolet Light as an anti-bacterial and anti-biofilm therapy is promising as UV light non-specifically targets DNA and RNA to assist in elimination of bacteria regardless of antibiotic resistance (Conner-Kerr et al., 1998). It plays a role in synthesis of cyclobutene pyrimidine dimers that disrupt cell growth and proliferation. The power of antibacterial photodynamic therapy (APDT) can be enhanced further through the use of photosensitizer molecules (PS), such as phenothiaziniums, tetrapyrroles, hypericin, and curcumin. Irradiation causes the electrons within a PS to enter higher energy orbitals. Upon return to ground state, these electrons can react with organic compounds inside cells, leading to free radical generation. These free radicals cause oxidative damage to the cell, promoting apoptosis (Ghorbani et al., 2018). It is unlikely that development of resistance to APDT would occur due to the non-specific nature of the target. Clinical application of this anti-bacterial method is limited to surface infections or medical device sterilization due to the difficulty of delivery and limited penetration of light through host-tissue (Argyraki et al., 2018). In addition, UV light is potentially carcinogenic to host-tissue, but has been shown to cause minimal damage when used at appropriate fluences (Barnes et al., 2018). More targeted treatment strategies utilizing light-based technology such as photodynamic therapy can further reduce host-tissue damage (Yin et al., 2013).

### 5.2 Antimicrobial peptides

Antimicrobial peptides (AMPs) have gained increasing attention due to their ability to decrease cell adhesion and reduce the thickness of a broad spectrum of biofilms (Shahrour et al., 2019). AMPs can be classified based on their secondary structure as either α-helical, β-sheet, loop, and extended peptides. To date, α-helical AMPs—such as Magainin-2 and LL-3—are the most well studied. The cationic amphipathic structure of these AMPs allows them to interact with negatively charged bacterial membranes, causing membrane lysis or invasion to carry out non-membranolytic mechanisms (Di Somma et al., 2020). AMPs exhibit additional antimicrobial activity as a result of non-membranolytic mechanisms, which are particularly useful in disruption genes or proteins that are essential for biofilm formation, function, and virulence (Luo and Song, 2021; Castillo-Juárez et al., 2022). There has been recent interest in isolating particular AMPs from plant essential oils. Eugenol derivatives from clove, bay, and pimento berry oils have been found to inhibit *Escherichia coli* O157:H7 biofilm formation by downregulating attachment proteins (Kim et al., 2016). Unfortunately, like antibiotics, AMPs are susceptible to intrinsic and acquired AMP resistance via various mechanisms, such as a more positively charged lipid membranes or efflux pumps—which may perpetuate selection for multi-drug resistant pathogens (Andersson et al., 2016).

### 5.3 Bacteriophage therapy

Additionally, bacteriophage therapy shows great promise as a specific, targeted option for treatment of biofilms, given their inherent antibacterial activity and minimal adverse effects. Bacteriophages are viruses that follow a lytic life cycle and infect specific strains of bacterial species, making them useful for targeting specific bacterial infections. Their lytic life cycle allows bacteriophages to replicate and spread through many bacteria, efficiently clearing infections. More importantly, the selective targeting of bacteria by bacteriophages spares human cells, thus, resulting in relatively few documented adverse events (Cesta et al., 2020). Due to coevolution with biofilm producing bacteria, bacteriophages have developed the ability to infect and lyse bacteria within biofilms through enzyme mediated degradation of biofilm ECM and can even infect cells during dormancy, causing lysis upon metabolic reactivation (Doub, 2020). Though bacteriophage resistance poses a challenge for therapy, bacteriophage “cocktails”—specific for multiple strains of a bacteria species—can be administered to reduce rates of resistance as well as help ensure the infecting pathogen is covered (Clarke et al., 2020). Furthermore, combination of phages and antibiotics has yielded promising results, even against multidrug-resistant biofilms (Akturk et al., 2019). In particular, pre-treatment of biofilms with phages has been shown to enhance the effects of antibiotics. (Townsend et al., 2020). Moreover, genetically engineered phages have also demonstrated the capacity of biofilm degradation and inhibitory effects (Li et al., 2020).

### 5.4 Immunotherapy

Several immunotherapeutic options have been explored with vaccination strategies and monoclonal antibodies being potential options. In the case of *S. aureus*, significant efforts have been made to develop a vaccine, but factors such as a lack of understanding of conserved antigens between strains and the need to account for both planktonic and biofilm components to fully eliminate infection have made a vaccine elusive (Bhattacharya et al., 2015). Monoclonal antibodies have had similar complications as preclinical and clinical trials fail to mitigate infection via passive immunity, however, application of monoclonal antibodies conjugated to antibiotics could provide another avenue for exploration as a way to concentrate antibiotics to the site of infection and increase their effectiveness (Speziale and Pietrocola, 2021).

## 6 Conclusion

As the average age of the US population increases, and the capacity of biomedical technology expands, so does the rate of hospitalization and surgical intervention (Pallin et al., 2014). Between 2005 and 2030, the number of total knee and total hip arthroplasties are predicted to increase by 174% (Antonelli and Chen, 2019). The number of artificial heart valve implantations is increasing by 5-7% every year (Saksena et al., 2019). These numbers only scratch the surface. Without urgent intervention, we can anticipate the rate of biofilm infections and antibiotic resistance to likewise multiply. Modern medicine is facing a microbial arms race, one which will require novel approaches, beyond conventional antibiotic therapy, to win. Inventions such as UV radiation, antimicrobial peptide design, phage therapy, and immunotherapy offer some possibilities to combat and control pathogenic biofilms and deserve further clinical investigation. Moreso, both public and private sector health entities would be wise to invest in both technology and training for clinicians involving biofilms. We are currently 20 years into the advent of antimicrobial stewardship programs and have deepened our understanding of microbial resistance and control (Charani and Holmes, 2019). By expanding these programs to explore biofilm regulation and resistance, medicine can enter the next generation of antimicrobial dominion to the benefit of patients worldwide.

## Author contributions

KG: Conceptualization, Investigation, Supervision, Writing – original draft, Writing – review & editing. JK: Conceptualization, Investigation, Writing – original draft, Writing – review & editing. DR: Conceptualization, Investigation, Writing – original draft, Writing – review & editing. MK: Conceptualization, Investigation, Writing – original draft, Writing – review & editing. AM: Conceptualization, Investigation, Writing – original draft, Writing – review & editing. PL: Writing – review & editing. AK: Writing – original draft. RS: Supervision, Writing – review & editing. YL: Conceptualization, Supervision, Writing – review & editing.

## References

[B1] Akturk E. Oliveira H. Santos S. B. Costa S. Kuyumcu S. Melo L. D. R.. (2019). Synergistic action of phage and antibiotics: parameters to enhance the killing efficacy against mono and dual-species biofilms. Antibiotics 8, 103. doi: 10.3390/antibiotics8030103 31349628 PMC6783858

[B2] An S.-Q. Murtagh J. Twomey K. B. Gupta M. K. O’Sullivan T. P. Ingram R.. (2019). Modulation of antibiotic sensitivity and biofilm formation in Pseudomonas aeruginosa by interspecies signal analogues. Nat. Commun. 10, 2334. doi: 10.1038/s41467-019-10271-4 31133642 PMC6536496

[B3] Andersson D. I. Hughes D. Kubicek-Sutherland J. Z. (2016). Mechanisms and consequences of bacterial resistance to antimicrobial peptides. Drug Resist. Update 26, 43–57. doi: 10.1016/J.DRUP.2016.04.002 27180309

[B4] Antonelli B. Chen A. F. (2019). Reducing the risk of infection after total joint arthroplasty: preoperative optimization. Arthroplasty 1, 4. doi: 10.1186/s42836-019-0003-7 35240760 PMC8787890

[B5] Argyraki A. Markvart M. Stavnsbjerg C. Kragh K. N. Ou Y. Bjørndal L.. (2018). UV light assisted antibiotics for eradication of *in vitro* biofilms. Sci. Rep. 8. doi: 10.1038/S41598-018-34340-8 PMC6218519 30397224

[B6] Aristotelous A. C. (2022). Biofilm neutrophils interactions under hypoxia: A mathematical modeling study. Math. Biosci. 352, 108893. doi: 10.1016/j.mbs.2022.108893 36029807

[B7] Asma S. T. Imre K. Morar A. Herman V. Acaroz U. Mukhtar H.. (2022). An overview of biofilm formation-combating strategies and mechanisms of action of antibiofilm agents. Life (Basel). 12. doi: 10.3390/life12081110 PMC9394423 35892912

[B8] Babin B. M. Atangcho L. van Eldijk M. B. Sweredoski M. J. Moradian A. Hess S.. (2017). Selective proteomic analysis of antibiotic-tolerant cellular subpopulations in *pseudomonas aeruginosa* biofilms. mBio 8. doi: 10.1128/mBio.01593-17 PMC5654934 29066549

[B9] Barnes J. L. Zubair M. John K. Poirier M. C. Martin F. L. (2018). Carcinogens and DNA damage. Biochem. Soc. Trans. 46, 1213–1224. doi: 10.1042/BST20180519 30287511 PMC6195640

[B10] Belfield K. Bayston R. Hajduk N. Levell G. Birchall J. P. Daniel M. (2017). Evaluation of combinations of putative anti-biofilm agents and antibiotics to eradicate biofilms of Staphylococcus aureus and Pseudomonas aeruginosa. J. Antimicrobial. Chemother. 72, 2531–2538. doi: 10.1093/jac/dkx192 28859444

[B11] Bernal-Mercado A. T. Gutierrez-Pacheco M. M. Encinas-Basurto D. Mata-Haro V. Lopez-Zavala A. A. Islas-Osuna M. A.. (2020). Synergistic mode of action of catechin, vanillic and protocatechuic acids to inhibit the adhesion of uropathogenic *Escherichia coli* on silicone surfaces. J. Appl. Microbiol. 128, 387–400. doi: 10.1111/jam.14472 31573730

[B12] Bhattacharya M. Wozniak D. J. Stoodley P. Hall-Stoodley L. (2015). Prevention and treatment of Staphylococcus aureus biofilms. Expert Rev. Anti Infect. Ther. 13, 1499–1516. doi: 10.1586/14787210.2015.1100533 26646248 PMC5142822

[B13] Cámara M. Green W. MacPhee C. E. Rakowska P. D. Raval R. Richardson M. C.. (2022). Economic significance of biofilms: a multidisciplinary and cross-sectoral challenge. NPJ Biofilms. Microbiomes. 8, 42. doi: 10.1038/s41522-022-00306-y 35618743 PMC9135682

[B14] Castillo-Juárez I. Blancas-Luciano B. E. García-Contreras R. Fernández-Presas A. M. (2022). Antimicrobial peptides properties beyond growth inhibition and bacterial killing. PeerJ 10. doi: 10.7717/PEERJ.12667 PMC8785659 35116194

[B15] Cesta N. Di Luca M. Corbellino M. Tavio M. Galli M. Andreoni M. (2020). Bacteriophage therapy: an overview and the position of Italian Society of Infectious and Tropical Diseases. Infez. Med. 28, 322–331. 32920567

[B16] Charani E. Holmes A. (2019). Antibiotic stewardship—Twenty years in the making. Antibiotics 8, 7. doi: 10.3390/antibiotics8010007 30678365 PMC6466570

[B17] Ciofu O. Moser C. Jensen P.Ø. Høiby N. (2022). Tolerance and resistance of microbial biofilms. Nat. Rev. Microbiol. 20, 621–635. doi: 10.1038/s41579-022-00682-4 35115704

[B18] Ciofu O. Rojo-Molinero E. Macià M. D. Oliver A. (2017). Antibiotic treatment of biofilm infections. APMIS 125, 304–319. doi: 10.1111/apm.12673 28407419

[B19] Clarke A. L. De Soir S. Jones J. D. (2020). The safety and efficacy of phage therapy for bone and joint infections: A systematic review. Antibiot. (Basel). 9, 1–11. doi: 10.3390/ANTIBIOTICS9110795 PMC7697170 33182795

[B20] Conner-Kerr T. A. Sullivan P. K. Gaillard J. Franklin M. E. Jones R. M. (1998). The effects of ultraviolet radiation on antibiotic-resistant bacteria *in vitro* . Ostomy. Wound Manage. 44, 50–56. 9866596

[B21] Diaconu O. Siriopol I. Poloşanu L. I. Grigoraş I. (2018). Endotracheal tube biofilm and its impact on the pathogenesis of ventilator-associated pneumonia. J. Crit. Care Med. 4, 50–55. doi: 10.2478/jccm-2018-0011 PMC6294989 30581995

[B22] Di Somma A. Moretta A. Canè C. Cirillo A. Duilio A. (2020). Antimicrobial and antibiofilm peptides. Biomolecules 10. doi: 10.3390/BIOM10040652 PMC7226136 32340301

[B23] Doub J. B. (2020). Bacteriophage therapy for clinical biofilm infections: parameters that influence treatment protocols and current treatment approaches. Antibiot. (Basel). 9, 1–12. doi: 10.3390/ANTIBIOTICS9110799 PMC7697957 33198058

[B24] Ghorbani J. Rahban D. Aghamiri S. Teymouri A. Bahador A. (2018). Photosensitizers in antibacterial photodynamic therapy: an overview. Laser. Ther. 27, 293–302. doi: 10.5978/islsm.27_18-RA-01 31182904 PMC6513767

[B25] Ghosh R. Barman S. Mandal N. C. (2019). Phosphate deficiency induced biofilm formation of Burkholderia on insoluble phosphate granules plays a pivotal role for maximum release of soluble phosphate. Sci. Rep. 9, 5477. doi: 10.1038/s41598-019-41726-9 30940828 PMC6445130

[B26] Gimza B. D. Larias M. I. Budny B. G. Shaw L. N. (2019). Mapping the global network of extracellular protease regulation in staphylococcus aureus. mSphere 4. doi: 10.1128/mSphere.00676-19 PMC6811363 31645429

[B27] Gominet M. Compain F. Beloin C. Lebeaux D. (2017). Central venous catheters and biofilms: where do we stand in 2017? APMIS 125, 365–375. doi: 10.1111/apm.12665 28407421

[B28] Hall C. W. Mah T.-F. (2017). Molecular mechanisms of biofilm-based antibiotic resistance and tolerance in pathogenic bacteria. FEMS Microbiol. Rev. 41, 276–301. doi: 10.1093/femsre/fux010 28369412

[B29] Hou J. Veeregowda D. H. van de Belt-Gritter B. Busscher H. J. van der Mei H. C. (2018). Extracellular Polymeric Matrix Production and Relaxation under Fluid Shear and Mechanical Pressure in Staphylococcus aureus Biofilms. Appl. Environ. Microbiol. 84. doi: 10.1128/AEM.01516-17 PMC5734043 29054874

[B30] Huigens R. W. (2018). The path to new halogenated quinolines with enhanced activities against staphylococcus epidermidis. Microbiol. Insights 11, 1178636118808532. doi: 10.1177/1178636118808532 30397386 PMC6207956

[B31] Ielapi N. Nicoletti E. Lorè C. Guasticchi G. Avenoso T. Barbetta A.. (2020). The role of biofilm in central venous catheter related bloodstream infections: evidence-based nursing and review of the literature. Rev. Recent Clin. Trials. 15, 22–27. doi: 10.2174/1574887114666191018144739 31656155

[B32] Jacqueline C. Caillon J. (2014). Impact of bacterial biofilm on the treatment of prosthetic joint infections. J. Antimicrobial. Chemother. 69, i37–i40. doi: 10.1093/jac/dku254 25135088

[B33] Jamal M. Ahmad W. Andleeb S. Jalil F. Imran M. Nawaz M. A.. (2018). Bacterial biofilm and associated infections. J. Chin. Med. Assoc. 81, 7–11. doi: 10.1016/j.jcma.2017.07.012 29042186

[B34] Jang I.-A. Kim J. Park W. (2016). Endogenous hydrogen peroxide increases biofilm formation by inducing exopolysaccharide production in Acinetobacter oleivorans DR1. Sci. Rep. 6, 21121. doi: 10.1038/srep21121 26884212 PMC4756669

[B35] Kim Y.-G. Lee J.-H. Gwon G. Kim S.-I. Park J. G. Lee J. (2016). Essential oils and eugenols inhibit biofilm formation and the virulence of escherichia coli O157:H7. Sci. Rep. 6, 36377. doi: 10.1038/srep36377 27808174 PMC5093407

[B36] Kirketerp-Møller K. Stewart P. S. Bjarnsholt T. (2020). The zone model: A conceptual model for understanding the microenvironment of chronic wound infection. Wound Repair Regener. 28, 593–599. doi: 10.1111/wrr.12841 PMC7540265 32529778

[B37] Kvist M. Hancock V. Klemm P. (2008). Inactivation of efflux pumps abolishes bacterial biofilm formation. Appl. Environ. Microbiol. 74, 7376–7382. doi: 10.1128/AEM.01310-08 18836028 PMC2592912

[B38] Lamret F. Varin-Simon J. Velard F. Terryn C. Mongaret C. Colin M.. (2021). Staphylococcus aureus strain-dependent biofilm formation in bone-like environment. Front. Microbiol. 12. doi: 10.3389/fmicb.2021.714994 PMC8453086 34557170

[B39] Lee H. W. Kharel S. Loo S. C. J. (2022). Lipid-coated hybrid nanoparticles for enhanced bacterial biofilm penetration and antibiofilm efficacy. ACS Omega. 7, 35814–35824. doi: 10.1021/acsomega.2c04008 36249378 PMC9558607

[B40] Li M. Shi D. Li Y. Xiao Y. Chen M. Chen L.. (2020). Recombination of T4-like Phages and Its Activity against Pathogenic Escherichia coli in Planktonic and Biofilm Forms. Virol. Sin. 35, 651–661. doi: 10.1007/s12250-020-00233-2 32451882 PMC7736419

[B41] Liu S. Chen L. Wang L. Zhou B. Ye D. Zheng X.. (2022). Cluster differences in antibiotic resistance, biofilm formation, mobility, and virulence of clinical enterobacter cloacae complex. Front. Microbiol. 13. doi: 10.3389/fmicb.2022.814831 PMC9019753 35464993

[B42] Liu J. Prindle A. Humphries J. Gabalda-Sagarra M. Asally M. Lee D. D.. (2015). Metabolic co-dependence gives rise to collective oscillations within biofilms. Nature 523, 550–554. doi: 10.1038/nature14660 26200335 PMC4862617

[B43] Liu J. Zhang J. Guo L. Zhao W. Hu X. Wei X. (2017). Inactivation of a putative efflux pump (LmrB) in *Streptococcus mutans* results in altered biofilm structure and increased exopolysaccharide synthesis: implications for biofilm resistance. Biofouling 33, 481–493. doi: 10.1080/08927014.2017.1323206 28587519

[B44] Luo Y. Song Y. (2021). Mechanism of antimicrobial peptides: antimicrobial, anti-inflammatory and antibiofilm activities. Int. J. Mol. Sci. 22. doi: 10.3390/IJMS222111401 PMC8584040 34768832

[B45] Malone M. Bjarnsholt T. McBain A. J. James G. A. Stoodley P. Leaper D.. (2017). The prevalence of biofilms in chronic wounds: a systematic review and meta-analysis of published data. J. Wound Care 26, 20–25. doi: 10.12968/jowc.2017.26.1.20 28103163

[B46] Metcalf D. Bowler P. (2013). Biofilm delays wound healing: A review of the evidence. Burns. Trauma 1, 5. doi: 10.4103/2321-3868.113329 27574616 PMC4994495

[B47] Miyaue S. Suzuki E. Komiyama Y. Kondo Y. Morikawa M. Maeda S. (2018). Bacterial memory of persisters: bacterial persister cells can retain their phenotype for days or weeks after withdrawal from colony–biofilm culture. Front. Microbiol. 9. doi: 10.3389/fmicb.2018.01396 PMC6028600 29997606

[B48] Namgoong S. Jung S.-Y. Han S.-K. Kim A.-R. Dhong E.-S. (2020). Clinical experience with surgical debridement and simultaneous meshed skin grafts in treating biofilm-associated infection: an exploratory retrospective pilot study. J. Plast. Surg. Handb. Surg. 54, 47–54. doi: 10.1080/2000656X.2019.1673170 31575315

[B49] Nishino K. Yamasaki S. Nakashima R. Zwama M. Hayashi-Nishino M. (2021). Function and inhibitory mechanisms of multidrug efflux pumps. Front. Microbiol. 12. doi: 10.3389/fmicb.2021.737288 PMC8678522 34925258

[B50] Olsen I. (2015). Biofilm-specific antibiotic tolerance and resistance. Eur. J. Clin. Microbiol. Infect. Dis. 34, 877–886. doi: 10.1007/s10096-015-2323-z 25630538

[B51] Ousey K. Ovens L. (2023). Comparing methods of debridement for removing biofilm in hard-to-heal wounds. J. Wound Care 32, S4–S10. doi: 10.12968/jowc.2023.32.Sup3b.S4 36971485

[B52] Overhage J. Campisano A. Bains M. Torfs E. C. W. Rehm B. H. A. Hancock R. E. W. (2008). Human host defense peptide LL-37 prevents bacterial biofilm formation. Infect. Immun. 76, 4176–4182. doi: 10.1128/IAI.00318-08 18591225 PMC2519444

[B53] Pallin D. J. Espinola J. A. Camargo C. A. (2014). US population aging and demand for inpatient services. J. Hosp. Med. 9, 193–196. doi: 10.1002/jhm.2145 24464735

[B54] Panlilio H. Rice C. V. (2021). The role of extracellular DNA in the formation, architecture, stability, and treatment of bacterial biofilms. Biotechnol. Bioeng. 118, 2129–2141. doi: 10.1002/bit.27760 33748946 PMC8667714

[B55] Perry E. K. Tan M.-W. (2023). Bacterial biofilms in the human body: prevalence and impacts on health and disease. Front. Cell Infect. Microbiol. 13. doi: 10.3389/fcimb.2023.1237164 PMC10499362 37712058

[B56] Pinto R. M. Soares F. A. Reis S. Nunes C. Van Dijck P. (2020). Innovative strategies toward the disassembly of the EPS matrix in bacterial biofilms. Front. Microbiol. 11. doi: 10.3389/fmicb.2020.00952 PMC7264105 32528433

[B57] Preda V. G. Săndulescu O. (2019). Communication is the key: biofilms, quorum sensing, formation and prevention. Discoveries. (Craiova). 7, e100. doi: 10.15190/d.2019.13 32309618 PMC7086079

[B58] Rather M. A. Gupta K. Mandal M. (2021). Microbial biofilm: formation, architecture, antibiotic resistance, and control strategies. Braz. J. Microbiol. 52, 1701–1718. doi: 10.1007/s42770-021-00624-x 34558029 PMC8578483

[B59] Rodríguez-Merchán E. C. Davidson D. J. Liddle A. D. (2021). Recent strategies to combat infections from biofilm-forming bacteria on orthopaedic implants. Int. J. Mol. Sci. 22, 10243. doi: 10.3390/ijms221910243 34638591 PMC8549706

[B60] Saksena D. Mishra Y. K. Muralidharan S. Kanhere V. Srivastava P. Srivastava C. P. (2019). Follow-up and management of valvular heart disease patients with prosthetic valve: a clinical practice guideline for Indian scenario. Indian J. Thorac. Cardiovasc. Surg. 35, 3–44. doi: 10.1007/s12055-019-00789-z PMC7525528 33061064

[B61] Santos A. P. A. Watanabe E. de Andrade D. (2011). Biofilme em marca-passo artificial: ficção ou realidade? Arq. Bras. Cardiol. 97, e113–e120. doi: 10.1590/S0066-782X2011001400018 22189616

[B62] Saverina E. A. Frolov N. A. Kamanina O. A. Arlyapov V. A. Vereshchagin A. N. Ananikov V. P. (2023). From antibacterial to antibiofilm targeting: an emerging paradigm shift in the development of quaternary ammonium compounds (QACs). ACS Infect. Dis. 9, 394–422. doi: 10.1021/acsinfecdis.2c00469 36790073

[B63] Schaible B. Taylor C. T. Schaffer K. (2012). Hypoxia Increases Antibiotic Resistance in Pseudomonas aeruginosa through Altering the Composition of Multidrug Efflux Pumps. Antimicrob. Agents Chemother. 56, 2114–2118. doi: 10.1128/AAC.05574-11 22290986 PMC3318321

[B64] Shahrour H. Ferrer-Espada R. Dandache I. Bárcena-Varela S. Sánchez-Gómez S. Chokr A.. (2019). AMPs as anti-biofilm agents for human therapy and prophylaxis. (Singapore: Springer), 257–279. doi: 10.1007/978-981-13-3588-4_14 30980362

[B65] Sharma D. Misba L. Khan A. U. (2019). Antibiotics versus biofilm: an emerging battleground in microbial communities. Antimicrob. Resist. Infect. Control. 8, 76. doi: 10.1186/s13756-019-0533-3 31131107 PMC6524306

[B66] Singh R. Sahore S. Kaur P. Rani A. Ray P. (2016). Penetration barrier contributes to bacterial biofilm-associated resistance against only select antibiotics, and exhibits genus-, strain- and antibiotic-specific differences. Pathog. Dis. 74, ftw056. doi: 10.1093/femspd/ftw056 27402781

[B67] Speziale P. Pietrocola G. (2021). Monoclonal antibodies targeting surface-exposed and secreted proteins from staphylococci. Vaccines (Basel). 9. doi: 10.3390/VACCINES9050459 PMC8147999 34064471

[B68] Tande A. J. Patel R. (2014). Prosthetic joint infection. Clin. Microbiol. Rev. 27, 302–345. doi: 10.1128/CMR.00111-13 24696437 PMC3993098

[B69] Townsend E. M. Moat J. Jameson E. (2020). CAUTI’s next top model - Model dependent Klebsiella biofilm inhibition by bacteriophages and antimicrobials. Biofilm 2, 100038. doi: 10.1016/j.bioflm.2020.100038 33381752 PMC7762788

[B70] Toyofuku M. Inaba T. Kiyokawa T. Obana N. Yawata Y. Nomura N. (2016). Environmental factors that shape biofilm formation. Biosci. Biotechnol. Biochem. 80, 7–12. doi: 10.1080/09168451.2015.1058701 26103134

[B71] Tran D. L. Huang R.-W. Chiu E. S. Rajhathy E. M. Gregory J. H. Ayello E. A.. (2023). Debridement: technical considerations and treatment options for the interprofessional team. Adv. Skin. Wound Care 36, 180–187. doi: 10.1097/01.ASW.0000920660.07232.f7 36940374

[B72] Vazquez-Rodriguez A. Vasto-Anzaldo X. G. Barboza Perez D. Vázquez-Garza E. Chapoy-Villanueva H. García-Rivas G.. (2018). Microbial Competition of Rhodotorula mucilaginosa UANL-001L and E. coli increase biosynthesis of Non-Toxic Exopolysaccharide with Applications as a Wide-Spectrum Antimicrobial. Sci. Rep. 8, 798. doi: 10.1038/s41598-017-17908-8 29335484 PMC5768876

[B73] Wakharde A. A. Halbandge S. D. Phule D. B. Karuppayil S. M. (2018). Anticancer drugs as antibiofilm agents in *candida albicans*: potential targets. Assay. Drug Dev. Technol. 16, 232–246. doi: 10.1089/adt.2017.826 29446984

[B74] Wang Y. Liu B. Grenier D. Yi L. (2019). Regulatory mechanisms of the luxS/AI-2 system and bacterial resistance. Antimicrob. Agents Chemother. 63. doi: 10.1128/AAC.01186-19 PMC6761564 31383657

[B75] Wu Y. Klapper I. Stewart P. S. (2018). Hypoxia arising from concerted oxygen consumption by neutrophils and microorganisms in biofilms. Pathog. Dis. 76. doi: 10.1093/femspd/fty043 PMC6454447 29688319

[B76] Yang B. Lei Z. Zhao Y. Ahmed S. Wang C. Zhang S.. (2017). Combination susceptibility testing of common antimicrobials *in vitro* and the effects of sub-MIC of antimicrobials on staphylococcus aureus biofilm formation. Front. Microbiol. 8. doi: 10.3389/fmicb.2017.02125 PMC5671985 29163415

[B77] Yasir M. Willcox M. D. P. Dutta D. (2018). Action of antimicrobial peptides against bacterial biofilms. Mater. (Basel). 11. doi: 10.3390/ma11122468 PMC6317029 30563067

[B78] Yin R. Dai T. Avci P. Jorge A. E. S. De Melo W. C. M. A. Vecchio D.. (2013). Light based anti-infectives: ultraviolet C irradiation, photodynamic therapy, blue light, and beyond. Curr. Opin. Pharmacol. 13, 731–762. doi: 10.1016/J.COPH.2013.08.009 24060701 PMC3831650

[B79] Zimmermann S. Klinger-Strobel M. Bohnert J. A. Wendler S. Rödel J. Pletz M. W.. (2019). Clinically approved drugs inhibit the staphylococcus aureus multidrug norA efflux pump and reduce biofilm formation. Front. Microbiol. 10. doi: 10.3389/fmicb.2019.02762 PMC6901667 31849901

